# Omega and Reverse Omega Incisions: Techniques for Immediate or Delayed Reconstruction of Mildly Ptotic and Larger Breasts Following Nipple-Sparing Mastectomy

**DOI:** 10.1007/s00266-025-05391-w

**Published:** 2026-01-26

**Authors:** Ilias Petrou, Dimitra Limnatitou, Andreas Vassiliou

**Affiliations:** 1 School of Medicine, European University Cyprus, 2404 Nicosia, Cyprus; 2 Department of Plastic and Reconstructive Surgery, Nicosia General Hospital, 2031 Nicosia, Cyprus

**Keywords:** Nipple-sparing mastectomy (NSM), Breast reconstruction, Breast-conserving therapy (BCT), Breast cancer, Ptotic breasts, Large breasts

## Abstract

**Background:**

Breast-Conserving Therapy (BCT) has become a widely accepted treatment option for selected breast cancer cases. Nipple-Sparing Mastectomy (NSM) is considered an effective and oncologically safe approach for certain patients, as it preserves the native skin and nipple, allowing for one- or two-stage reconstruction with optimal cosmetic outcomes. This study aimed to provide our department’s experience utilising the Omega and Reverse Omega incision techniques for immediate or delayed breast reconstruction for patients who had undergone NSM.

**Method:**

Our study included sixteen cancer patients with mildly ptotic and large breasts who underwent immediate or delayed breast reconstruction using prosthetic implants following NSM, where the Omega incision was utilised, either bilaterally or unilaterally, from January 2022 to December 2024, at the Plastic and Reconstructive Surgery Department of Nicosia General Hospital. Patient demographics, co-morbidities, pre- and postoperative breast anthropometric measurements, and surgical complications were recorded and analysed.

**Results:**

Sixteen patients underwent a total of twenty-one nipple-sparing mastectomy procedures for either breast cancer (90.47%) or risk reduction (9.52%), followed by breast reconstruction. The average age at the time of reconstruction was 64.4 years. The mean preoperative body mass index was 26.73 kg/m^2^, and 25% were smokers. The mean follow-up was 11.62 months. From the patients who had undergone NSM, 12 (75%) were in the immediate reconstruction group, while the other 4 (25%) belonged to the delayed reconstruction group. Unilateral breast reconstruction was performed in 11 (52.38%) breasts. Bilateral NSM and immediate reconstruction with pre-pectoral implants was performed in 10 (47.61%) breasts. The Reverse Omega technique was performed in seven breasts. Single-stage breast reconstruction was performed for 17 (80.95%) breasts, while two-stage reconstruction for 4 (19.05%). Complications were reported, including a case of haematoma 1 (4.76%) and three cases of implant loss (14.28%).

**Conclusion:**

The Omega incision appears to be a safe and effective technique for immediate or delayed reconstruction for selected cases of NSM eligible cancer patients with slightly ptotic and/or larger breasts.

**Level of Evidence IV:**

Evidence obtained from multiple time series with or without the intervention, such as case studies. Dramatic results in uncontrolled trials might also be regarded as this type of evidence.

## Introduction and Background

According to the American Cancer Society, the estimated number of female breast cancer cases in the USA for 2023 was 297,790, with approximately 43,170 fatalities attributed to the disease [1]. In Cyprus, breast cancer was reported as the most common cancer in women according to a 2023 report, accounting for 34% of new cases, while the European Union average was 29% [2]. As part of the management, Breast-Conserving Therapy (BCT) has become an established surgical treatment option, where feasible, by offering improved quality of life for patients and the potential for optimal aesthetic outcomes [3–6]. Geoffrey Keynes was the first physician to report positive outcomes with BCT in 1937 [7]. Subsequent studies, such as the clinical trial by Atkins et al. 1972, had further supported the efficacy of BCT combined with radiotherapy, demonstrating comparable overall survival results to mastectomy for early-stage breast cancer [8].

Nipple-Sparing Mastectomy (NSM) had emerged as a technique aimed at preserving the Nipple–Areolar Complex (NAC) and the skin envelope [9–11]. In 1962, Freeman had initially proposed the use of the sub-mammary incision to preserve the nipple during breast surgery for benign lesions [12]. It was later described that, for patients undergoing prophylactic mastectomy, there was no significant difference in risk reduction between individuals with retained or excised nipples [13, 14]. In the following years, NSM had gained popularity due to its potential to improve patient quality of life, more favourable cosmetic outcomes and facilitation of immediate breast reconstruction with oncological safety comparable to non-NSM in selected cases [10, 11, 15, 16].

Careful patient selection for NSM candidates was advised, with factors such as macromastia and moderate to severe breast ptosis warranting consideration, due to the increased risk of complications such as flap and nipple necrosis, infection, and potentially suboptimal aesthetic outcomes. According to Tousimis et al., ideal NSM candidates typically exhibited minimal or no breast ptosis (grade 0 or 1), had a cup size of A or B and a body mass index (BMI) of less than 30 kg/m^2^ [10].

Several techniques have been described for skin-reducing NAC-sparing mastectomy for large and ptotic breasts. Various combinations of incisions and pedicles have been outlined to achieve the objectives for skin-reducing NAC-sparing mastectomy, which include: ensuring adequate oncologic clearance with an easy mastectomy access point, maintaining vascularity of the nipple–areola complex, optimising skin envelope reduction, improving breast contour, and creating a well-defined pocket suitable for immediate reconstruction [17–19].

Incision patterns described for NAC-sparing skin-reducing mastectomy include the dual-pedicle, three-pedicle, and four-pedicle approaches. The wide base bipedicled (WIBB) flap is a dual-pedicle, nipple-preserving flap supplied by superior/medial and inferior perforators used in most large/ptotic breasts. In contrast, the three-pedicle-based nipple-sparing skin-reducing mastectomy (TP-NSSRM) preserves three separate vascular pedicles—superior, inferior, and medial or lateral—and is often used for aggressive skin reduction (larger, drooping, and high-risk/previously operated breasts) to further enhance NAC perfusion. Lastly, with the four-pedicle approach, the NAC is preserved with four separate pedicles, including the superior/medial, inferior, lateral and superolateral, or medial. Thus, blood supply to the NAC is maximised from all quadrants with the aim to minimise ischaemic risk in extremely large, ptotic, or irradiated/operated breasts 20–22].

All these techniques are based on preservation of vascular pedicles, mainly derived from the perforators of the internal mammary artery, supplemented by contributions from the lateral thoracic and thoracoacromial arteries, as well as anterior intercostal perforators in inferiorly based designs, thus avoiding periareolar incisions [20–22]. These techniques can be used in both immediate and two-stage reconstruction with expanders.

Currently, there are limited data in the literature regarding reconstructive outcomes following nipple-sparing mastectomy. Our proposed techniques, the Omega and Reverse Omega incisions, which will be described in detail in following sections, would allow for correction of mildly ptotic and/or larger breasts during or after NSM, for patients considered eligible for this type of mastectomy from an oncological perspective, aiming at optimal cosmetic outcomes while still maintaining the desired oncological results. Whether implemented immediately or in a delayed setting following NSM, it would permit breast reconstruction in both mildly ptotic and larger breasts, yielding favourable cosmetic results. The purpose of this study was to provide our clinic’s experience and evaluate the efficacy and safety of Omega and Reverse Omega incisions as reliable surgical approaches for breast reconstruction of mildly ptotic and larger breasts during or after NSM.

## Materials and Methods

This study was conducted in the Plastic and Reconstructive Surgery Department at Nicosia General Hospital from January 2022 to December 2024. It included sixteen early-stage breast cancer patients with mildly ptotic and/or larger breasts who underwent NSM followed by breast reconstruction using prosthetic implants and utilising either the Omega or the Reverse Omega incision techniques.

Patients eligible for inclusion in this study were of female gender assigned at birth, with an early breast cancer diagnosis. All included cases were deemed suitable for NSM and either early or delayed reconstruction, unilateral or bilateral, with or without symmetrisation, during the time period of our data collection. Detailed breast anthropometric measurements were obtained in different stages of the study, including the selection stage, to determine eligible cases. Grade of breast ptosis was not used as an inclusion or exclusion criterion. Instead, patient selection was carried out on a case-by-case assessment based on anthropometric measurements. Those with a suprasternal notch to nipple distance (SN-N) of 20–27 cm were identified as suitable candidates for the Omega incision technique (incision superior to the NAC). For patients with nipple to inframammary fold (N-IMF) distance of 8–12 cm, the Reverse Omega incision (incision inferior to the NAC) was considered suitable. For patients with both of those distances in the ranges stated above, the choice between the Omega incision or the Reverse Omega incision was made at the discretion of the operating surgeon, with the aim to correct the distance that was most divergent from the desired range, taking into consideration the individual patient anatomy and preferences. Smoking status and Diabetes Mellitus (DM) were not strictly classed as exclusion criteria, however, as these were considered higher risk patients groups, the decision regarding type of operation was thoroughly discussed with them preoperatively. Exclusion criteria for this study included patients not fulfilling the above inclusion criteria or not wishing to participate in the study or not wishing to undergo the surgical intervention in question. All surgical interventions for the cases included in this study were carried out at Nicosia General Hospital.

Data were collected prospectively and included patient demographics such as age and gender, individual traits such as preoperative weight, height, BMI, and smoking status, as well as clinical details about the surgical interventions that took place for each case. Breast anthropometric measurements were recorded both pre- and postoperatively, including the SN-N distance, the N-IMF distance, and the breast base width (BBW). Complications were also recorded and included in the results. The pre- and postoperative plans were thoroughly discussed with all patients, who were regularly followed up at the hospital’s outpatient breast clinic.

### Ethical Considerations and Patient Consent

All procedures performed in this study were by the ethical standards of the institutional and/or national research committee and with the 1964 Helsinki Declaration and its later amendments or comparable ethical standards. After explaining the purpose and nature of the study, informed consent was obtained by individuals wishing to participate. The authors ensured and protected the anonymity and confidentiality of the participants throughout the process of this study.

### Data Collection and Statistical Analysis

Data collection and fundamental statistical analysis were conducted using Microsoft Excel sheets. Descriptive statistics (mean ± SD) were employed to express continuous variables, while frequencies and percentages were used for categorical variables.

## Technique Description

**Fig. 1:**
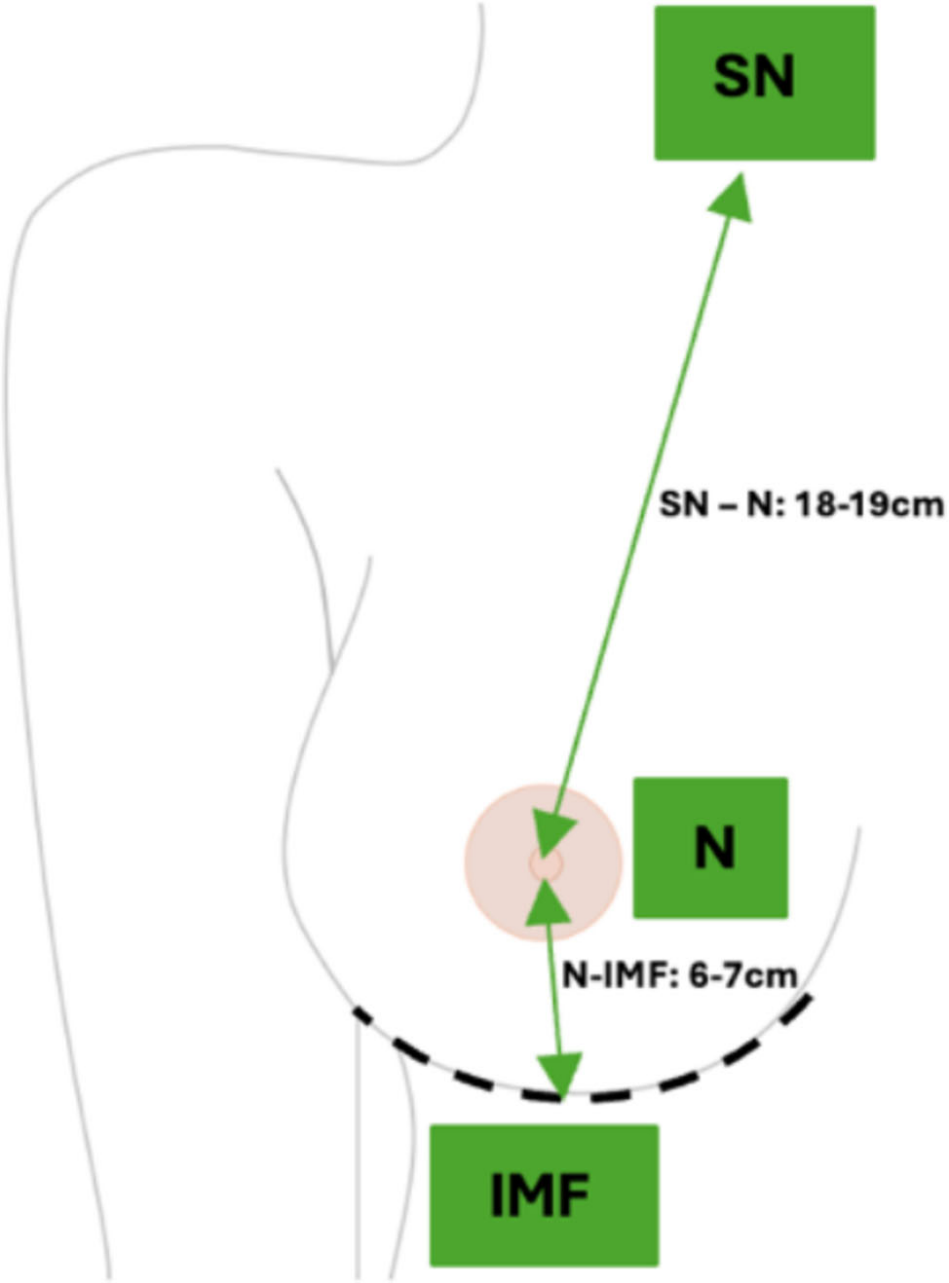
Desired breast measurements for SN-N and N-IMF

The purpose of the Omega and the Reverse Omega incisions was to reduce the SN-N distance or the N-IMF distance, respectively, while providing an access point for performing NSM and reconstruction, in order to enhance cosmetic outcomes. The desired breast measurements for SN-N and N-IMF are demonstrated in Figure 1.

As mentioned above, breast anthropometric measurements, including the SN-N, the N-IMF, and the BBW, were measured for all patients, from the initial surgical intervention planning stage. Subsequent assessments and clinic visits were conducted using our clinic’s specific breast protocol*.*

**Fig. 2:**
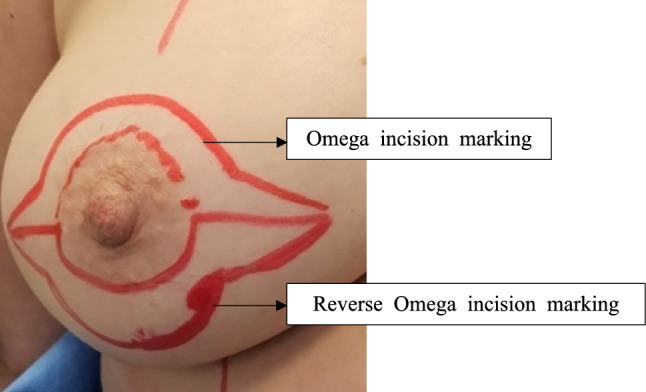
Demonstration of Omega and Reverse Omega incision markings in real life. In the cases discussed in this paper, either the former or the latter were used, not a combination. The figure serves to demonstrate the markings for both incisions in real life, but they have not been used concurrently. As mentioned above, the choice between the two would be made by the operating Plastic Surgeon according to the distance requiring correction based on the breast anthropometric measurements and individual patient anatomy and wishes

SN-N, N-IMF, and BBW were again measured for all patients on the day of surgery by the operating Plastic Surgeon. An Omega-shaped outline would be drawn either above or below the NAC. The decision as to whether the incision would be above or below the areola would be made on a case-by-case basis, depending on the SN-N and N-IMF distances, as well as the oncological aims of the operation. When performed above the nipple, this technique would allow for the correction of mildly ptotic breasts by lifting the NAC. Conversely, when performed below the nipple, it would address an increased distance been the nipple to the inframammary fold, resulting in more aesthetically pleasing outcomes. Whether implemented immediately or during delayed reconstruction following NSM, Omega or Reverse Omega incision method would yield better cosmetic outcomes for either mildly ptotic or larger breasts (Table 1, Fig. 2).

**Table 1:** Omega and Reverse Omega incisions

(a) Omega incision	The choice between Omega incision or Reverse Omega incision was determined preoperatively by the operating Plastic Surgeon according to preoperative measurements on the day of surgery.
	Patients were measured and incision lines were drawn in the upright position by the operating Plastic Surgeon before the start of the operation.
(b) Reverse Omega incision	The solid lines in Images (a) & (b) represent the fixed parts of the incision, i.e. a semi-circular line encircling either the top or bottom half of the nipple with up to 2 cm horizontal line extensions on both sides.
	The dotted lines in Images (a) & (b) represent the variable parts of the incision, i.e. distance x is determined by the operating Plastic Surgeon according to the degree of correction required for either the SN-N distance or the N-IMF distance.

**Fig. 3:**
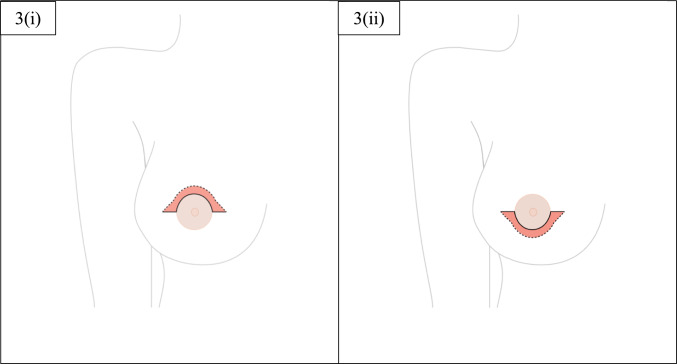
These images demonstrate the areas of skin removed in order to provide an access point for the NSM and reconstruction stages. Image 3(i) represents the wound following an Omega incision and Image (ii) represents the wound following a Reverse Omega incision

Following the marking of the incision lines, the patients were transferred to the operating theatre for commencement of the surgical intervention. All operations were carried out under general anaesthesia, with the patient in the supine position and both arms in a 45-degree angle. All cases were performed jointly by General Surgery and Plastic Surgery teams. The procedure would start with the incision following the pre-determined Omega or Reverse Omega drawing, and the entire block of enclosed skin would be removed in order to provide an access point for the NSM, as well as for creating the plane for either breast implant or tissue expander placement (Fig. 3).

Once the mastectomy part was completed by the General Surgery team, the reconstruction part would commence by the Plastic Surgery team. For the cases discussed in this study, single-stage reconstruction was performed, with the use of breast implants, for 17 breasts, and two-stage reconstruction was carried out for four breasts, with the initial placement of tissue expanders. All breast implants used for primary reconstruction in this study were round silicone implants, placed either above the pectoralis major muscle (pre-pectoral) along with titanised mesh or below (sub-pectoral). Suitable size was determined based on BBW distance, individual patient anatomy, overlying skin quality, Plastic Surgeon recommendation and patient wishes. Regarding tissue expanders, placement was below the pectoralis major muscle (sub-pectoral). The decision between single- or two-stage reconstruction was made preoperatively, taking into consideration factors such as breast skin quality, plans for adjuvant treatment following NSM, Plastic Surgeon recommendations, and patient wishes.

**Fig. 4:**
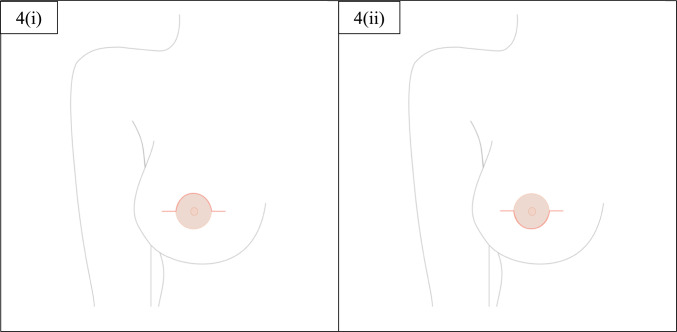
Final wound appearance at the end of the operation for 4(i) Omega incision and for 4(ii) Reverse Omega incision

**Fig. 5:**
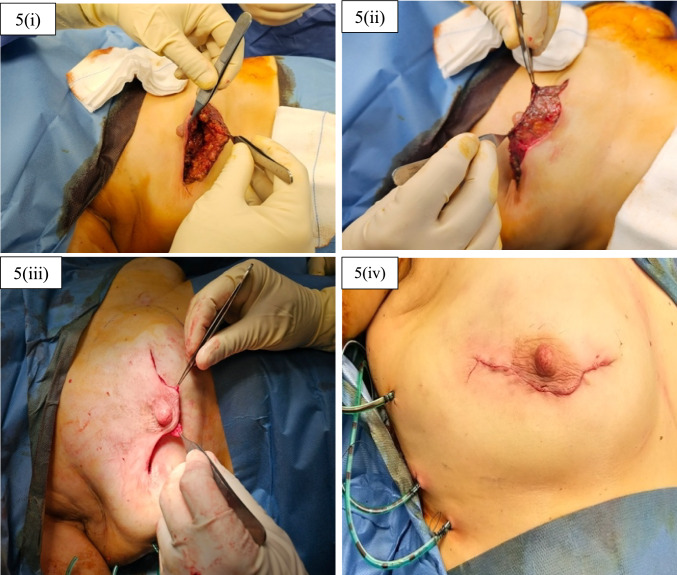
Wound closure steps; 5(i) Reverse Omega incision wound; 5(ii) and 5(iii) remaining superficial breast fascia and soft tissues providing extra layer between skin and prosthetic material; and 5(iv) final wound after fascia, dermis, and skin closure

For all cases discussed in this study, drains were placed at the end of the operation. Wounds were closed with 2-0 PDS single interrupted dermal sutures for approximation and 3-0 Monocryl continuous subcuticular sutures for skin closure. Any remaining superficial breast fascia at the wound edges would be used as another layer between implant or expander and dermis for added protection of the prosthetic material and would be sutured with 2-0 PDS. The final result at the end of the operation would be a semi-circular wound line with two horizontal extensions on either side, as demonstrated in the figures below (Figs. 4, 5).

All patients included in the study had received intraoperative antibiotic coverage with an intravenous first-generation cephalosporin, which was continued postoperatively for two additional doses. Analgesia was tailored to each individual’s needs and was continued upon discharge with over-the-counter analgesics taken as required. Patients were discharged 48 hours postoperatively, with or without drains in situ, depending on the output. Drains were removed once the output, after the 2nd postoperative day and following mobilisation of the patient, were less than 30 millilitres per 24 hours.

Following surgery, patients were advised to wear a compression bra daily, with the exception of shower time, for a minimum of 2 weeks. Following that initial postoperative period, a transition bra was recommended for daily use for an additional time period of 4 weeks. After 6 weeks, patients were advised that they could wear any bra, without an underwire. Long-term bra usage, 3 months postoperatively, was tailored based on each individual’s healing progress and preferences. Patients were advised that they could shower 3 days following surgery, abstain from driving for at least a week postoperatively and until they felt safe to perform an emergency stop without discomfort or pain and avoid lifting any weights for at least 4 weeks. Surgical dressing changes were scheduled at intervals of 3, 7, and 14 days postoperatively, while follow-up visits were initiated at intervals of 3, 7, 14, 30, 90, 180, and lastly, 360 days.

## Results

The study included 16 patients with a mean age of 64.4 years (range, 56–71 years). Amongst six patients in whom the Omega or Reverse Omega incision was not performed, the inferior dermal flap technique was employed. The mean body mass index was 26.73 (range, 21.1–35.1) and 25% of patients were smokers (Table 2). The mean follow-up was 11.62 months. Of patients who had undergone nipple-sparing mastectomy, 12 (75%) were in the immediate reconstruction group, while the other 4 (25%) in the delayed reconstruction group. The total number of breasts for NSM reconstructions was 21. Unilateral breast reconstruction was carried out in 11 (52.38%) breasts. In seven breasts, unilateral NSM was performed along with immediate pre-pectoral (five breasts) or sub-pectoral (two breasts) implant-based reconstruction. Unilateral NSM with delayed reconstruction using a sub-pectoral tissue expander was carried out in four breasts. Bilateral NSM with immediate pre-pectoral implant-based reconstruction was performed in 10 (47.61%) breasts. The Reverse Omega technique was performed in seven breasts. In the context of pre-pectoral implant placement (15 breasts), titanised mesh implants had also been utilised.

**Table 2:** Summary data consecutive nipple-sparing mastectomy reconstructions

	Value (%)
*Patient population*	
No. of patients	16
NSM reconstructions	21
Total reconstructions for analysis	21
*Follow-up period, months*	
Mean ± SD	11.62 ± 1.5
Median	12
*Demographics and risk factors*	
Age, yr	
Mean ± SD	64.4 ± 4.08
Range	56–71
*Preoperative weight, kg*	
Mean ± SD	69.62 ± 9.14
Range	54–88
*Height, cm*	
Mean + SD	1.61 ± 6.59
Range	1.52-1.76
*BMI, kg/m*^*2*^	
Mean	26.73 ± 4.38
Range	21.1–35.1
Smoking	4/16 (25%)
Diabetes mellitus	0/16 (0%)
Prior personal history of breast cancer	2/16 (12.5%)
Prior family history of breast cancer	6/16 (37.5%)
*Implant placement*	
Pre-pectoral	15*/21
Sub-pectoral	2/21
Implant volume, cc	17/21
*Left*	
Mean	309.28cc
Range	225–425cc
Right	
Mean	333.50cc
Range	275–400cc
Tissue expander placement (pre or sub pec)	4/21
*End tissue expander fill volume, cc (final volume before stage 2)*	
Mean	325cc
Range	280–360cc
*Indication*	
Prophylactic	2/21 (9.52%)
Therapeutic	19/21 (90.47%)
*Laterality*	
Unilateral	11/21 (52.38%)
Bilateral	10/21 (47.61%)
*Mastectomy incision*	
Omega	14/21 (66.66%)
Reverse Omega	7/21 (33.33%)
*Other prosthetics*	
Titanised mesh	15*/21 (71.42%)

^*^For all pre-pectoral implants, titanised mesh was also used

The mean preoperative right SN-N distance was 22.81 ± 2.31, while the mean postoperative was 20.87 ± 2.27. For the left breast, the mean preoperative SN-N distance was 23 ± 2.33, while the mean postoperative was 21.43 ± 2.55. The mean preoperative right N-IMF distance was 7.93 ± 1.69, while the mean postoperative distance was 7.25 ± 1.18. The mean preoperative N-IMF distance for the left breast was 8.06 ± 1.69, while the mean postoperative was 7.62 ± 1.31. All postoperative measurements would be referring to the numbers obtained at the 12-month follow-up. Detailed patients’ breast anthropometric measurements pre- and postoperatively are illustrated in Table 3, while Table 4 illustrates patients’ breast anthropometric measurements stratified by Incision Type (Omega vs. Reverse Omega) and Laterality.

**Table 3:** Patient’s breast anthropometric measurements (overall)

	SN-N	N-IMF	BBW
*Right Breast*			
Range preoperative (cm)	20–27	6–12	9–6
Mean preoperative (cm) ± SD	22.81 ± 2.31	7.93 ± 1.69	12.56 ± 1.71
Range postoperative (cm) at 12-month follow-up	17–27	6–10	9–16
Mean postoperative (cm) at 12-month follow-up ± SD	20.87 ± 2.27	7.25 ± 1.18	12.56 ± 1.71
*Left Breast*			
Range preoperative (cm)	20–27	6–12	9–16
Mean preoperative (cm) ± SD	23 ± 2.33	8.06 ± 1.69	12.56 ± 1.71
Range postoperative (cm) at 12-month follow-up	17–26	6–10	9–16
Mean postoperative (cm) at 12-month follow-up ± SD	21.43 ± 2.55	7.62 ± 1.31	12.56 ± 1.71

**Table 4:** Patient’s breast anthropometric measurements, stratified by incision type (Omega vs Reverse Omega) and laterality

	SN-N	N-IMF	BBW
*Omega Incision*			
Right Breast			
Range preoperative (cm)	20–27	6–11	9–16
Mean preoperative (cm) ± SD	23.5 ± 2.23	7.66 ± 1.49	12.91 ± 1.78
Range postoperative (cm) at 12-month follow-up	18–27	6–9	9–16
Mean postoperative (cm) at 12-month follow-up ± SD	21.16 ± 2.24	7.08 ± 0.99	12.91 ± 1.78
Left Breast			
Range preoperative (cm)	20–27	6–11	9 - 16
Mean preoperative (cm) ± SD	23.66 ± 2.26	7.83 ± 1.52	12.91 ± 1.78
Range postoperative (cm) at 12-month follow-up	17–26	6–10	9–16
Mean postoperative (cm) at 12-month follow-up ± SD	21.91 ± 2.50	7.5 ± 1.24	12.91 ± 1.78
*Reverse Omega Incision*			
Right Breast			
Range preoperative (cm)	20–21	7–12	11–13
Mean preoperative (cm) ± SD	20.75 ± 0.95	8.75 ± 2.21	11.5 ± 1
Range postoperative (cm) at 12-month follow-up	17–22	6–10	11–13
Mean postoperative (cm) at 12-month follow-up ± SD	20 ± 2.44	7.75 ± 1.70	11.5 ± 1
Left Breast			
Range preoperative (cm)	20–22	8–12	11–13
Mean preoperative (cm) ± SD	21 ± 1.15	8.75 ± 2.21	11.5 ± 1
Range postoperative (cm) at 12-month follow-up	17–22	6–10	11–13
Mean postoperative (cm) at 12-month follow-up ± SD	20 ± 2.44	8 ± 1.63	11.5 ± 1

**Fig. 6:**
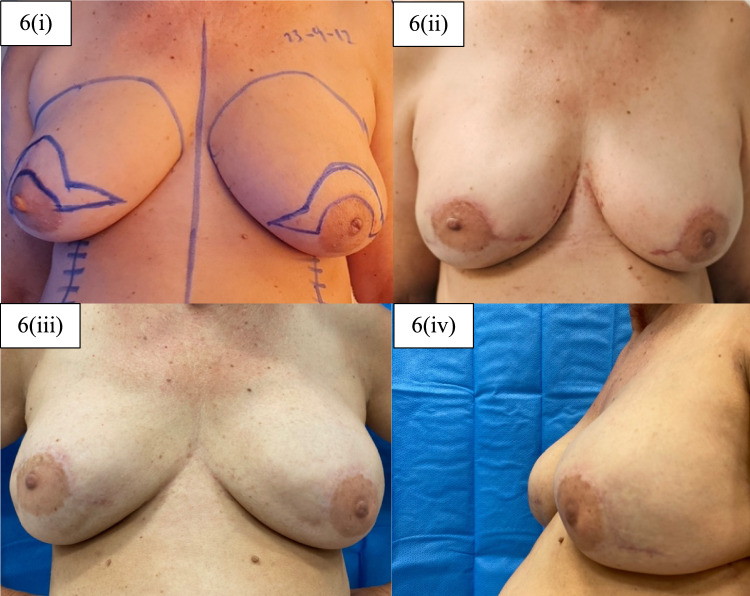
Patient before and after surgery. 6(i) Patient presented with initial sternal notch-nipple distance (SN-N) of 23 cm and with nipple-inframammary fold distance (N-IMF) of 9 cm on both breasts’ sides. 6(ii-iv) Long -term follow-up after bilateral NSM and immediate implant-based pre-pectoral breast reconstruction with Omega incision and the use of titanised meshes. Patient’s postoperative SN-N was at 21 cm and N-IMF at 8 cm on both breasts’ sides

**Fig. 7:**
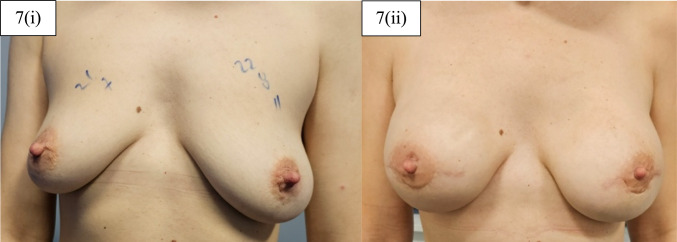
Patient before and after surgery. 7(i) Patient presented with initial sternal notch-nipple distance (SN-N) of 22 cm on the left side and 21 cm on the right side and with nipple-inframammary fold distance (N-IMF) of 8 cm on the left side and 7 cm on the right side. 7(ii) Long-term follow-up after bilateral NSM and immediate implant-based pre-pectoral breast reconstruction with Reverse Omega incision and the use of titanised meshes. Patient’s postoperative SN-N was at 22 cm and N-IMF at 6 cm on both breasts’ sides

Type of reconstruction and complications are demonstrated in Table 5. Single-stage breast reconstruction was performed for 17 (80.95%) breasts, while two-stage reconstruction for 4 (19.05%). The overall complication rate was calculated at 19.05%, although they were only observed in the single-stage reconstruction group (23.53%). Amongst the documented complications, for one breast, it was a postoperative haematoma, for another breast, it was implant loss with no specification in the records as to the nature of the cause, and for another skin necrosis of both breasts on a single patient. It was noted that, out of those cases, the former two patients were non-smokers, whereas the latter were a smoker. Figures 6 and 7 demonstrate the breast appearances of two patients before and after surgery.

**Table 5:** Type of reconstruction and complications for the total (21 breasts) nipple-sparing mastectomy reconstructions

Reconstruction	No. of breasts (%)
Single-stage (implant)	17/21 (80.95%)
Two-stage (tissue expander/implant)	4/21 (19.05%)

Complications (overall)	No. of breasts (%)
Haematoma	1/21 (4.76%)
Implant loss—unspecified	1/21 (4.76%)
Implant loss—skin necrosis	2/21 (9.52%)
Complications (per reconstruction group)	
Complications documented for single-stage reconstruction (implant)	4/17 (23.53%)
Complications documented for two-stage reconstruction (tissue expander/implant)	0/4 (0.00%)

## Discussion

Various techniques had been proposed in the literature regarding the reconstruction of mildly ptotic and/or larger breasts following mastectomy.

In this study, the mean age of participants was 64.4 years, which is higher than the 48.3 years reported by Cordova et al., likely due to differences in patient selection, as our cohort primarily comprised older individuals undergoing therapeutic mastectomy. Furthermore, the patients’ mean preoperative body mass index (BMI) was 26.73 kg/m^2^, and 25% of patients were active smokers, findings comparable to those of Cordova et al. (BMI 24.80 kg/m^2^, 21.6% smokers), indicating similar baseline risk profiles and supporting the generalisability of our results. Notably, none of our patients had diabetes mellitus, warranting further study in that subgroup [17].

Postoperative complications have been reported with varying frequencies in the literature [17–19], [22]. In our series, complications included one case of postoperative haematoma (1 breast; 4.76% overall; 5.88% of the single-stage reconstruction group) and three cases of implant loss (3 breasts; 14.28% overall; 17.6% of the single-stage reconstruction group). The overall complication rate in our cohort was 19.04% (all complications occurring in the single-stage reconstruction group) which is comparable to the 15.5% reported by Cordova et al [17]. In their study, minor complications consisted of partial-thickness skin necrosis (8.5%), partial-thickness NAC necrosis (2.8%), and periprosthetic seroma (2.8%), while major complications included infection and implant loss in one patient (1.4%). Furthermore, a systematic review by Endara et al. demonstrated that complication rates vary significantly according to the incision type used for NSM [22]. In particular, transareolar incisions were associated with a high risk of necrosis (81.82%), whereas periareolar/circumareolar incisions carried a considerably lower risk (17.81%), which is consistent with the findings of our study. The lowest risk for necrosis was found in the group with the mastopexy procedure.

Additionally, in a recent systematic review, it was reported that the overall complication rate for NSM with either immediate or delayed reconstruction was 29.08%, with higher rates in the former group compared to the latter (37.52% vs 14.8%). In addition, partial or complete NAC necrosis, as well as skin flap necrosis rates had also been reported in the literature as higher in the single-stage reconstruction group. For our sample group, as already mentioned, complications included a postoperative haematoma and three cases of implant loss. Out of those three cases, for one breast, the reason for the implant loss was unspecified, whereas for the other two breasts, it was due to skin necrosis. Both skin necrosis cases had occurred on the same patient, and there was a positive social history of smoking.

Multiple techniques had been examined in terms of reconstructive options post-NSM, including direct placement of implant, two-stage placement of expander to implant and autologous tissue transfer. Skin-reducing techniques such as vertical mastopexy, Wise-pattern mastopexy, supra-areolar Batwing skin excision, as well as circumareolar incisions have all been described and compared, mostly in regard to complications [23]. It was noted that the supra-areolar skin excision seemed to have higher percentage of NAC necrosis compared to other methods in a study (12.5% partial and 18.7% total), although the sample size was small (eight patients and 16 breasts), while some of the other techniques had been investigated for larger populations [23], [24].

It was also noted that all patients experiencing total or partial NAC necrosis were smokers, and for two out of the three individuals suffering this complication, the necrosis was bilateral [24]. For the eight patients of the supra-areolar Batwing incision study, inclusion criteria were described as individuals who were candidates for either prophylactic or therapeutic NSM, with grade II or grade III ptosis. For the reconstruction, either tissue expanders were immediately placed, or deep inferior epigastric perforator flaps (DIEP) were performed, and the use of acellular dermal matrixes was also noted. Patients from both reconstruction groups had suffered NAC necrosis; two breasts from the DIEP group and three breasts from the expander group [24].

In another study, the Batwing incision with immediate implant placement was compared to standard implant-based reconstruction, with no significant differences in terms of haematoma or seroma formation, necrosis of NAC or skin, and major infection, while the former allowed for a favourable cosmetic outcome in patients with ptotic or larger breasts. It was noted that the rate of minor infections was slightly higher in the Batwing group [25]. When compared to Wise pattern, for upper pole breast tumours, Batwing incision mastopexy seemed to have lower complication rates, less favourable aesthetic results, with, however, higher patient satisfaction. The parameters for evaluating cosmetic results, as well as the overall satisfaction, were determined by the patients [26]. For individuals with significant ptosis, Batwing incision was compared to both Wise incision with dermal infolding and Wise pattern with inferior dermal flap, and was found to have smaller rates of NAC necrosis compared to the latter (11.1% in the Batwing group and 43.8% in the Wise pattern with inferior dermal flap group), and slightly higher rate compared to the former (10% for the Wise incision and dermal infolding group) [27]. For breast cancer patients with macromastia, Batwing incision was found to result in short operative times, low complication rates, favourable aesthetic outcomes, and high patient satisfaction [28].

Summarily, our proposed technique allows not only for NAC elevation but also for its descent, thereby providing greater flexibility in repositioning according to individual patient needs. Many of our patients presented with mild breast ptosis and explicitly requested correction. With the Omega incision, we were able to achieve up to 2 cm of NAC ascent, which would not have been feasible with other techniques like the simple linear incision. From an aesthetic perspective, patients considered a periareolar scar more acceptable, as it can be concealed by underwear or swimwear, whereas lateral scars are often more visible. Lastly, breast surgeons performing the mastectomies have also expressed a preference for the Omega incision, as it facilitates surgical exposure and access.

That being said, we fully acknowledge that each technique has its own advantages and limitations. In our practice, all available options are discussed with patients prior to surgery, and the Omega or Reverse Omega incision is selected only when deemed most appropriate. Our intention is not to replace established approaches but rather to contribute an additional tool to the reconstructive armamentarium. While our 12-month follow-up has shown reassuring results in terms of both objective measurements and aesthetic outcomes, we agree that further studies with larger cohorts and longer follow-up will be essential to confirm long-term stability, patient satisfaction, and to directly compare outcomes with other techniques.

## Limitations and Future Implications

While this study could offer an insight into the potential usability of the Omega and Reverse Omega incisions for certain breast cancer cases where NSM is an option, limitations should be pointed out, and future implications and recommendations should be discussed.

While this technique might appear promising in terms of complication rates, risks such as infection, haematoma or seroma formation, NAC or skin necrosis, and implant loss would require thorough exploration through further studies with larger sample sizes and further comparison to other methods. The Omega/Reverse Omega incision techniques could potentially enhance patient’s quality of life by providing natural and aesthetically pleasing results, which is something that would also warrant further investigation through further research, with validated tools, for measurable and quantifiable results. Furthermore, in our study, the follow-up period would not allow for assessing longer term results through the years in terms of satisfaction, durability, and late complications. Additionally, since none of our included patients had a medical history of diabetes mellitus, outcomes in this group of patients needs to be further studied. Further research into patient-reported outcomes and quality of life post-surgery would be critical for evaluating the psychosocial impact, patient satisfaction, and complication rates, in both shorter and longer timescales.

Additionally, future studies could potentially compare the Omega/Reverse Omega incision techniques to other reconstructive options, in order to determine specific advantages and disadvantages across various patient populations. The techniques we had described might not be suitable for all patients, particularly individuals with significant macromastia or complex breast anatomy; therefore, further research would be useful in terms of determining and refining eligibility criteria. Lastly, we recognise the absence of control groups as a limitation of our study. While control groups are commonly used in experimental research to establish causal relationships, our research employed an observational method, limiting our ability to control confounding variables.

As more data emerges, future research could develop standardised protocols for patient selection, surgical techniques, and postoperative care, enhancing its reproducibility and safety. Despite these limitations, we believe that our study design enabled us to capture real-world clinical experiences, which are valuable for establishing optimal practices in the clinical setting.

## Conclusion

Omega and Reverse Omega incision techniques could potentially become effective options for reconstruction following NSM in selected patients with mild ptosis or larger breasts respectively. More research with larger number of cases would be required to determine more accurately the safety, efficacy, and patient satisfaction of these methods compared to other techniques, as well as potentially establish the specific parameters for patients who would benefit the most from them.

## Data Availability

The data used and analysed in this study are available from the corresponding author on reasonable request.

## Abbreviations

BBW: Breast base width
BCT: Breast-conserving therapy
BMI: Body mass index
DIEP: Deep inferior epigastric artery perforator
NAC: Nipple–areolar complex
NSM: Nipple-sparing mastectomy
N-IMF: Nipple to inframammary fold
IMF: Inframammary fold
SD: Standard deviation
SN-N: Suprasternal notch to nipple
